# Coarse particulate matter (PM10) induce an inflammatory response through the NLRP3 activation

**DOI:** 10.1186/s12950-024-00388-9

**Published:** 2024-05-02

**Authors:** Damariz Marín-Palma, Jorge H. Tabares-Guevara, Natalia Taborda, Maria T. Rugeles, Juan C. Hernandez

**Affiliations:** 1 Infettare, Facultad de Medicina, Universidad Cooperativa de Colombia, Medellín, Colombia; 2 Grupo Inmunovirología, Facultad de Medicina, Universidad de Antioquia UdeA, Medellín, Colombia; 3 Grupo de Investigaciones Biomédicas Uniremington, Programa de Medicina, Facultad de Ciencias de la Salud, Corporación Universitaria Remington, Medellín, Colombia

**Keywords:** PM10, Air pollution, Inflammatory response, NLRP3 inflammasome, Lung inflammation

## Abstract

**Introduction:**

PM exposure can induce inflammatory and oxidative responses; however, differences in these adverse effects have been reported depending on the chemical composition and size. Moreover, inflammatory mechanisms such as NLRP3 activation by PM10 have yet to be explored.

**Objective:**

To assess the impact of PM10 on cell cytotoxicity and the inflammatory response through in vitro and in vivo models.

**Methodology:**

Peripheral blood mononuclear cells (PBMCs) from healthy donors were exposed to PM10. Cytotoxicity was determined using the LDH assay; the expression of inflammasome components and the production of pro-inflammatory cytokines were quantified through qPCR and ELISA, respectively; and the formation of ASC complexes was examined using confocal microscopy. For in vivo analysis, male C57BL6 mice were intranasally challenged with PM10 and bronchoalveolar lavage fluid was collected to determine cell counts and quantification of pro-inflammatory cytokines by ELISA. RNA was extracted from lung tissue, and the gene expression of inflammatory mediators was quantified.

**Results:**

PM10 exposure induced significant cytotoxicity at concentrations over 100 µg/mL. Moreover, PM10 enhances the gene expression and release of pro-inflammatory cytokines in PBMCs, particularly IL-1β; and induces the formation of ASC complexes in a dose-dependent manner. In vivo, PM10 exposure led to cell recruitment to the lungs, which was characterized by a significant increase in polymorphonuclear cells compared to control animals. Furthermore, PM10 induces the expression of several inflammatory response-related genes, such as NLRP3, IL-1β and IL-18, within lung tissue.

**Conclusion:**

Briefly, PM10 exposure reduced the viability of primary cells and triggered an inflammatory response, involving NLRP3 inflammasome activation and the subsequent production of IL-1β. Moreover, PM10 induces the recruitment of cells to the lung and the expression of multiple cytokines; this phenomenon could contribute to epithelial damage and, thus to the development and exacerbation of respiratory diseases such as viral infections.

**Supplementary Information:**

The online version contains supplementary material available at 10.1186/s12950-024-00388-9.

## Introduction

Air pollution is related to about 6.7 million annual deaths and is considered a public health problem worldwide [1]. Factors such as high population density, fossil fuel usage, and industrial growth contribute to high levels of pollution [1]. Therefore, most of the population is exposed to harmful pollutants. Among these agents, particulate matter (PM) has been strongly linked to respiratory diseases such as asthma, lung cancer, chronic obstructive pulmonary disease (COPD), and recently, to increased susceptibility to viral infections [2–7].

In 2011, a study conducted in Japan involving 63,520 individuals found a higher incidence of respiratory diseases, particularly pneumonia, related to long-term exposure to PM [8]. Similarly, the results in Europe revealed a population hazard ratio of 1.55 (95% CI: 1.05–2.29) for developing lung adenocarcinoma with each increase of 5 µg/m^3^ in PM 2.5 [9]. Beyond its impact on the respiratory system, PM has been linked to cardiovascular, neurological diseases and even adverse effects on newborns [10–12].

Specifically, particulate matter is a mixture of suspended particles that can reach the airways and even enter systemic circulation, causing adverse reactions [13]. PM can be classified based on its aerodynamic diameter into three categories: PM10 (coarse), PM2.5 (fine), and PM0.1 (ultrafine). It has been reported that depending on its emission source, PM can vary on its chemical and size, and can induce different adverse effects [14, 15]. However, the main mechanisms described after PM exposure are inflammatory and oxidative responses.

Although the mechanisms induced by PM are unclear, it has been proposed that oxidative stress, tissue damage, genotoxicity, vasoconstriction, and inflammation may mediate the deleterious effects of PM [16–18]. In particular, the inflammatory response triggered by PM exposure has been associated with worsening respiratory diseases, including asthma and COPD [2, 6]. Inflammasomes have been identified as inflammatory components activated in response to pollutant exposure [19]. In this sense, Jia et al. reported that inhibition of the NLRP3 (NLR Family Pyrin Domain Containing 3) pathway prevents lung damage, decrease inflammatory response and cell number in the bronchoalveolar lavage fluid (BALF) following in vivo exposure to PM2.5 [20].

Similarly, it has been reported in human bronchial cells that cigarette smoke can induce pyroptosis, a cell death mechanism derived from the inflammasome pathway [21]. Likewise, it was observed that the stimuli with tobacco extract (CSE) and diesel exhaust particulates (DEPs) increase IL-1β secretion in lung tissues, and induce NLRP3 inflammasome activation [21]; however, the signals that trigger this relationship are still unknown.

Notably, most studies have focused on evaluating the effects of fine PM (PM2.5) and cigarette smoke due to their ability to penetrate the lower airways. However, it has been reported that PM10 can also induce an inflammatory response in airways [22]. This study aimed to assess the in vitro and in vivo inflammatory properties of PM10, focused on NLRP3 inflammasome activation.

## Materials and methods

### PM10 collection and stock preparation

Coarse particulate matter (PM10) was collected in Valle de Aburrá, Colombia, and obtained as previously described [23]. Briefly, 100 quartz filters were obtained and cut into small pieces, and stock suspensions were prepared in high-purity sterile water by sonication followed by lyophilization. To perform the assays, the PM10 stimuli were prepared in sterile water and stored at -20 °C until use.

### Isolation and stimulation of peripheral mononuclear blood cells (PBMCs)

Peripheral blood samples were obtained from healthy adult donors (males and females between 21 and 56 years old), with no history of smoking or pre-existing inflammatory conditions such as allergies or autoimmune diseases. In addition, individuals with symptoms of infectious diseases within weeks prior to sampling were excluded. Ficol-histopaque 1077 (Sigma-Aldrich Chemical Co., St. Louis, MO, USA) was used to isolate the PBMCs by gradient method. Then, PBMCs were seeded at a density of 250,000–2,000,000 in RPMI-1640 medium supplemented with 2% heat-inactivated fetal bovine serum (FBS), 2 mM L-glutamine and 1% penicillin-streptomycin (all reagents from Sigma-Aldrich). Cells were exposed to PM10 (1–400 µg/mL) and incubated for 2–24 h (to evaluate the best timing for gene expression of inflammatory mediators) at 37 °C with 5% CO_2_. As a positive control for cytotoxicity, Triton X-100 (5%) was used, while LPS (0.05 µg/mL; Sigma-Aldrich L4391-1MG) was used for cytokine assays. The cellular fractions and supernatants were collected by centrifuging at 730 x g for 10 min and stored at -20 °C until further use.

### Cytotoxicity assessed by lactate dehydrogenase (LDH)

Cell cytotoxicity was evaluated by the LDH colorimetric assay CyQUANT™ Kit (Invitrogen, Waltham, Massachusetts, USA) after 24 h of stimulation, following the manufacturer’s instructions. The reading was performed in a microplate reader (Multiskan™ FC Microplate Photometer, Thermo Scientific, Waltham, Massachusetts, USA) at 490 nm.

### Inhibition assay

The PBMCs were seeded at a density of 250,000 cells/well in RPMI-1640 medium (2% FBS, 1% penicillin-streptomycin and 2 mM L-glutamine) and treated with glibenclamide or curcumin (commercial formulas, Merck Laboratory and Coaspharma Laboratory, respectively) for 1 h at 37 °C with 5% CO_2_. Then, the PBMCs were exposed to PM10 (50 and 100 µg/mL) and incubated overnight. Cell supernatants were collected for ELISA.

### RNA extraction and cDNA synthesis

Total RNA was extracted from 2,000,000 cells/well with the Direct-zol RNA Miniprep kit (Zymo Research), according to the manufacturer’s recommendations. RNA purity and quantity were assessed by spectrophotometry at 260–280 nm (Nanodrop one, Thermo Fisher Scientific, USA). From 150ng of total RNA, cDNA was constructed using the iScript cDNA synthesis kit (Bio-Rad, USA).

### Real-time PCR (qPCR)

qPCR was performed to quantify the mRNA of the inflammatory response components, using SYBR Green qPCR Master mix kit (Thermo Fisher Scientific, Waltham, MA, USA) as previously reported [24]. Briefly, the qPCR conditions were standardized for each gene following an amplification protocol of 40 cycles (Table S1). The relative mRNA content of NLRP3, NLRP1 (NLR Family Pyrin Domain Containing 1), NLRC4 (NLR Family CARD Domain Containing 4), AIM2 (Absent In Melanoma 2), TXNIP (Thioredoxin Interacting Protein), ASC, Caspase-1, Caspase-5, IL-1β, IL-18, IL-36γ, IL-6, IL-8 and TNF-α were quantified; Phosphoglycerate kinase (PGK) gene expression was used to normalize the RNA content. For qPCR analysis, CFX Manager Version: 1.5.534.0511 software (Bio-Rad, Hercules, CA, USA) was used, and the ΔΔCt method was applied to calculate the relative gene expression levels.

### Cytokine quantification by ELISA

The PBMC supernatants were used to quantified IL-1β, IL-6, IL-8 and TNF-α levels after 24 h of PM10 exposure, by ELISA (#437,004, Biolegend, Thermo Fisher; #555,220, BD Biosciences, San Jose, CA, USA; #431,504 Biolegend, Thermo Fisher, and #88-7346-76 eBioscience, Thermo Fisher Scientific, respectively) as previously reported [24].

### ASC complex formation

THP-1 cells expressing green fluorescent protein (GFP) coupled to the ASC protein (THP-1-ASC-GFP) were seeded at a density of 10,000 cells/well in 96 well-plates, and using Dulbecco’s Modified Eagle Medium (DMEM, Sigma-Aldrich) with 10% of FBS for 24 h at 37 °C with 5% CO_2_. THP-1-ASC-GFP cells were primed with LPS (0.1 µg/mL) overnight and then exposed to increasing doses of PM10 for 1 h, 3 h and 5 h. Visualization was performed by confocal microscopy.

### in vivo model

Eight-week-old male C57BL6 mice obtained from Charles Rivers (Portage, MI, USA) were bred and housed at 22 ± 1 °C, with free access to food and water and maintained under a 12 h light/dark cycle. The in vivo assays were performed under specific pathogen-free conditions at the Universidad de Antioquia animal facility (Medellín, Colombia). Mice were treated daily intranasally with PM10 (10 and 100 µg/mL in PBS) or vehicle (PBS) for 5 days; for each dose, the final volume was 50µL. On day 6, the mice were euthanized via intraperitoneal overdose of ketamine/xylizane (100/10 mg/kg). In total, eighteen mice were included, six mice per treatment (PM10 or PBS).

The weights of the mice were monitored every day. After euthanasia, a cardiac puncture was performed to collect whole blood, and BALF was obtained by perfusion of the lungs with 1 mL of sterile PBS. Total cell count and differential count were performed by light microscopy and Wright’s stain, respectively, from BALF. For histopathological analysis, the left lobe of the lungs was fixed with paraformaldehyde for 48 h, embedded in paraffin, and stained with hematoxylin-eosin (Sigma Aldrich).

The lung’s right lobe and peribronchial nodule were lysed in TRIzol-Reagent. RNA extraction was performed with Direct-zol RNA Miniprep kit (Zymo Research, Orange, CA, USA), according to the manufacturer’s instructions. RNA quantification, cDNA construction and qPCR were performed as described in the previous section to quantify NLRP3, Caspase-1, IL-1β, IL-18, IL-6, CXCL1 and MUC5AC. Additionally, IL-6 levels in BALF and serum were quantified using ELISA kits (#431,301, Biolegend, Thermo Fisher).

### Statistical analysis

The data analysis was performed using GraphPad Prism 9.0.2 (GraphPad Software, CA, USA). Normality and homoscedasticity were assessed using the Shapiro-Wilk and Levene tests, respectively. Comparisons between two or more groups were made using parametric ANOVA or Kruskal-Wallis tests with a 95% confidence. In case of statistical differences, the post hoc tests HDS of Tukey and Dunn, respectively, were applied. Significant differences were considered with p-values were lower than 0.05. The data are presented as the median ± IQR (interquartile range).

### Ethics

All the participants enrolled in the study were adults, who read and signed an informed consent form. Te study was previously reviewed and approved by the research ethics committee from Universidad Cooperativa de Colombia (Act 003 / 2018). Additionally, the experiments were carried out in accordance with the Code of Ethics of the World Medical Association (Declaration of Helsinki). The animal experiments were carried out following international guidelines and by the animal ethics committee from Universidad de Antioquia (Act 117 / 2018).

## Results

### PM10 affects cell viability and induces an inflammatory response in PBMCs

**Fig. 1:**
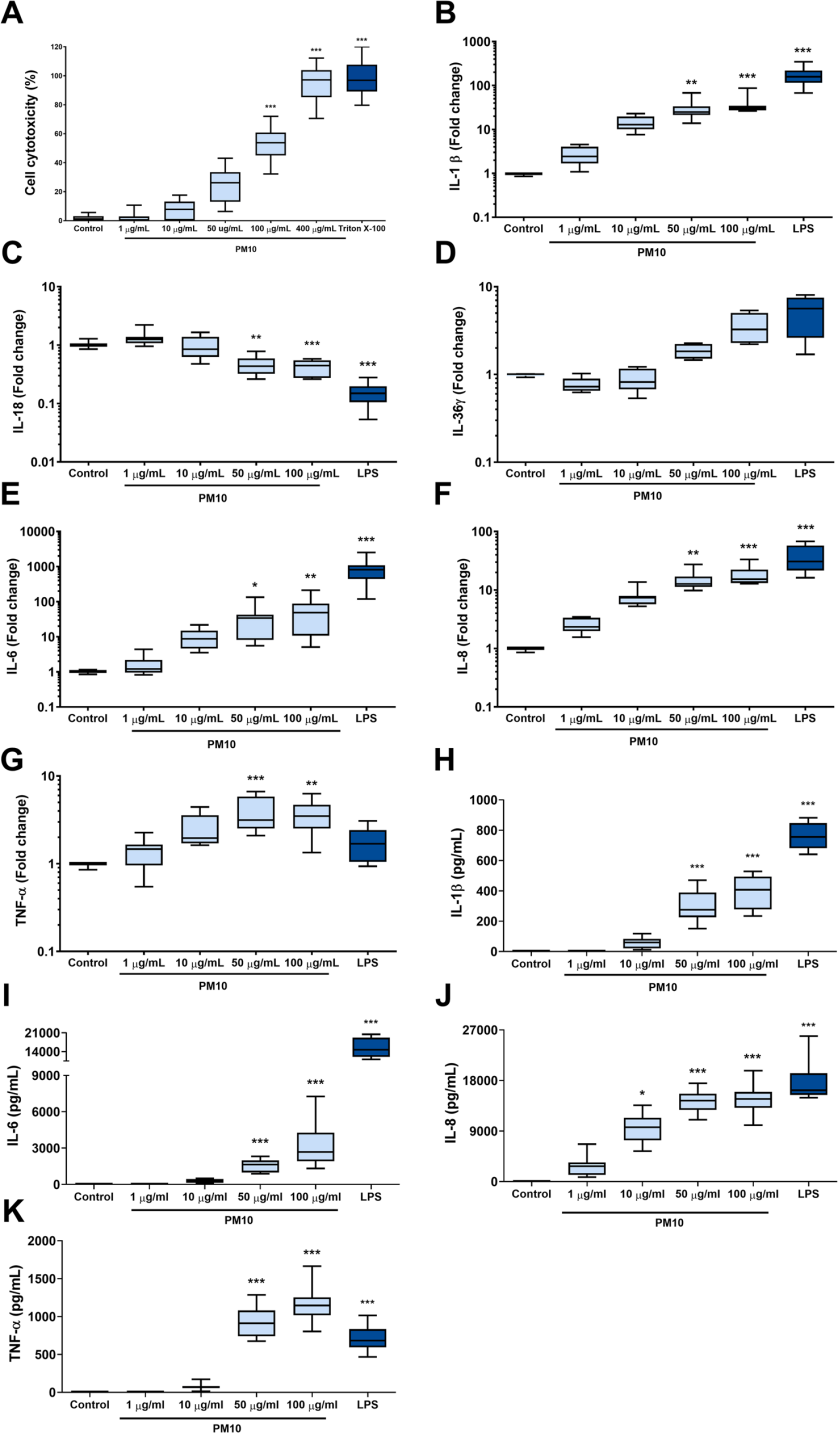
PM10 exposure induces cytotoxicity and the production of inflammatory mediators in PBMCs. Cellular cytotoxicity was assessed by LDH release in PBMCs exposed to increasing doses of PM10 (1–400 µg/mL) for 24 h. **(A)** Percentage of cytotoxicity in PBMCs exposed to PM10. Triton X-100 was used as a positive control; **(B-G)** Expression of pro-inflammatory cytokines quantified by qPCR (fold change) at 18 h of exposure. LPS (0.05 µg/mL) was used as a positive control **(H-K)** Production of pro-inflammatory cytokines from PBMCs exposed to PM10 (1–100 µg/mL) for 24 h and quantified by ELISA (pg/mL). Untreated cells were used as a negative control. Statistical comparisons were made using the Kruskal-Wallis test with a 95% confidence level. In case of statistical differences, Dunn’s HDS post hoc tests (or multiple benchmark) were applied (*n* = 6–8). Significant differences (comparisons with the control) are represented at the top of the bars (*p < 0.05; **p < 0.01; ***p < 0.001)

To evaluate the cytotoxic effect of PM10, an LDH assay was carried out after 24 h of exposure. An increase in LDH release was observed in PBMCs exposed to PM10 in a concentration-dependent manner, significantly over 100 µg/mL (Fig. 1A). The induction of the inflammatory response in primary cells was subsequently explored; and after the best timing for the gene expression of inflammatory mediators was determined, the cells were exposed to PM10 and incubated for 2–24 h at 37 °C (Figure S1). An increase after 18 h of exposure in the gene expression of IL-1β (*p* < 0.05), IL-6 (*p* < 0.05), IL-8 (*p* < 0.05) and TNF-α (*p* < 0.01) was observed when PMBCs were exposed to up to 50 µg/mL PM10 (Fig. 1B, E, F and G, respectively). No significant differences were observed in IL-36γ (*p* < 0.05) expression (Fig. 1D). Interestingly, a decrease in the mRNA expression of IL-18 was observed in cells treated with 50–100 µg/mL PM10 (*p* < 0.001) (Fig. 1C).

Additionally, increased production of the pro-inflammatory cytokines IL-1β (*p* < 0.001), IL-6 (*p* < 0.001), IL-8 (*p* < 0.05) and TNF-α (*p* < 0.001) was observed after 24 h of PM10 exposure (Fig. 1H-K).

### PM10 exposure induces changes in the expression of inflammasome-related genes and the formation of ASC specks

**Fig. 2:**
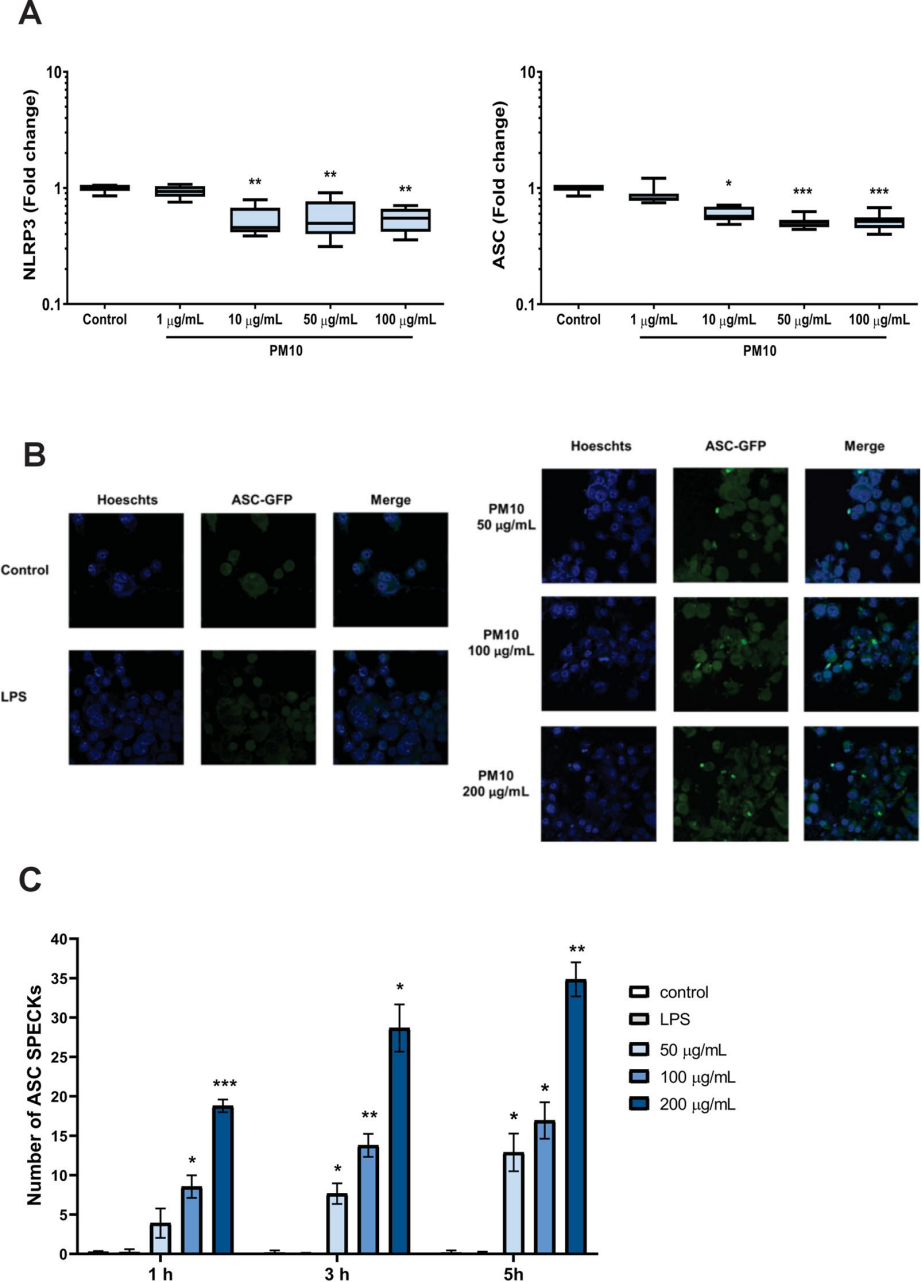
PM10 alters inflammasome component expression and induces ASC complexes assembly. The gene expression of inflammasome components in PBMCs exposed to PM10 was quantified by qPCR. The formation of ASC complexes in THP-1 cells expressing ASC-GFP and exposed to increasing doses of PM10 (50–200 µg/mL) was evaluated by confocal microscopy at different times (1–5 h). **(A)** Expression of NLRP3 and ASC (fold change); **(B)** Representative image of ASC specks by confocal microscopy. **(C)** the number of ASC specks was calculated as the average of 6 fields with the 40X objective, and LPS was used as the priming signal. The data are presented as median ± IQR (*n* = 3). Statistical comparison was made using Kruskal-Wallis tests with a confidence level of 95%, and Dunn’s HDS post hoc (or multiple benchmark) tests were performed. Significant differences are represented at the top of the bars (*p < 0.05; *p* < 0.01; ***p < 0.001)

Since the gene expression and release of IL-1β were observed in cells exposed to PM10, inflammasome-related genes were assessed. Notably, decreased expression of NLRP3 and ASC (both over 10 µg/mL; *p* < 0.05) were observed (Fig. 2A). Additionally, decreases in NLRC4 and Caspase-5 were observed at 100 µg/mL. No significant differences were observed for NLRP1, AIM2, TXNIP and Caspase-1 (Figure S2). Furthermore, ASC speck formation was evaluated by microscopy as an indicator of inflammasome activation. ASC specks were observed upon PM10 stimulation in a concentration-dependent manner (Fig. 2B and C).

**Fig. 3:**
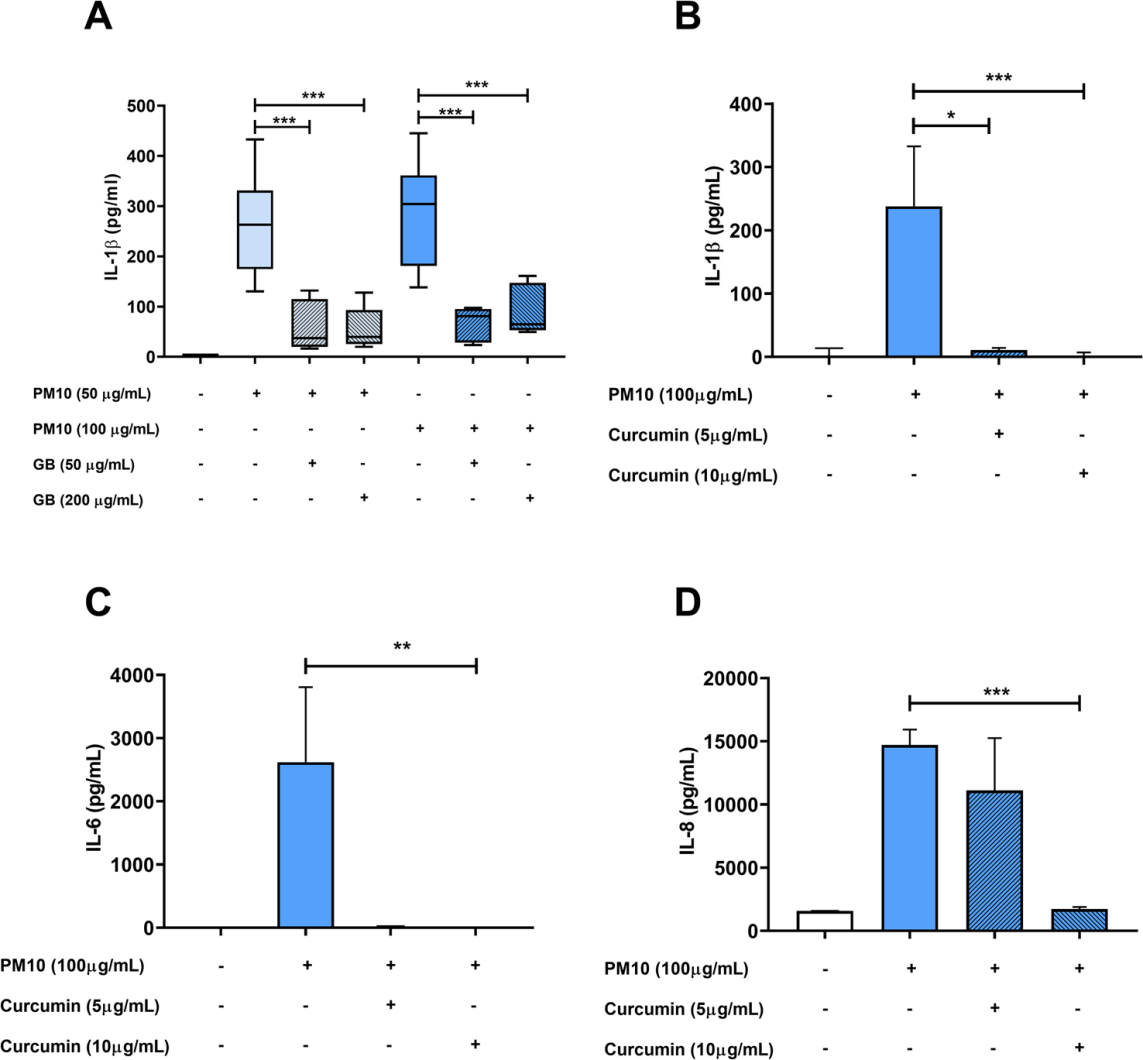
Glibenclamide and curcumin decrease IL-1β production in PBMCs exposed to PM10. IL-1β production was evaluated by ELISA in PBMCs supernatants treated with glibenclamide (GB) or curcumin and exposed to PM10 (100 µg/mL) for 24 h. Results are presented as pg/mL of **(A)** IL-1β in the presence of GB, **(B)** IL-1β in the presence of curcumin, and **(C)** IL-6 and **(D)** IL-8 in the presence of curcumin. Data are presented as median ± IQR (*n* = 3–4). Statistical comparison was made using Kruskal-Wallis tests with a confidence level of 95%, and Dunn’s HDS post hoc (or multiple benchmark) tests were performed. Significant differences are represented at the top of the bars (*p < 0.05; **p < 0.01; ***p < 0.001)

Because K + efflux is a crucial process for inflammasome activation and IL-1β release, to assess its role in the PM10 response, cells were treated with glibenclamide, an inhibitor of K + efflux. A significant decrease in IL-1β production was observed in cells exposed to PM10 and treated with 50–200 µg/mL of glibenclamide (*p* < 0.001; Fig. 3A).

Additionally, curcumin has been reported to inhibit NLRP3 activation by preventing K + efflux, ASC oligomerization and speck formation; therefore, the effect of curcumin was evaluated in cells exposed to PM10. Curcumin inhibited the production of IL-1β, IL-6 and IL-8 in cells exposed to PM10 (*p* < 0.001; Fig. 3B and D, respectively).

### PM10 induces the recruitment of cells to the airways of mice

**Fig. 4:**
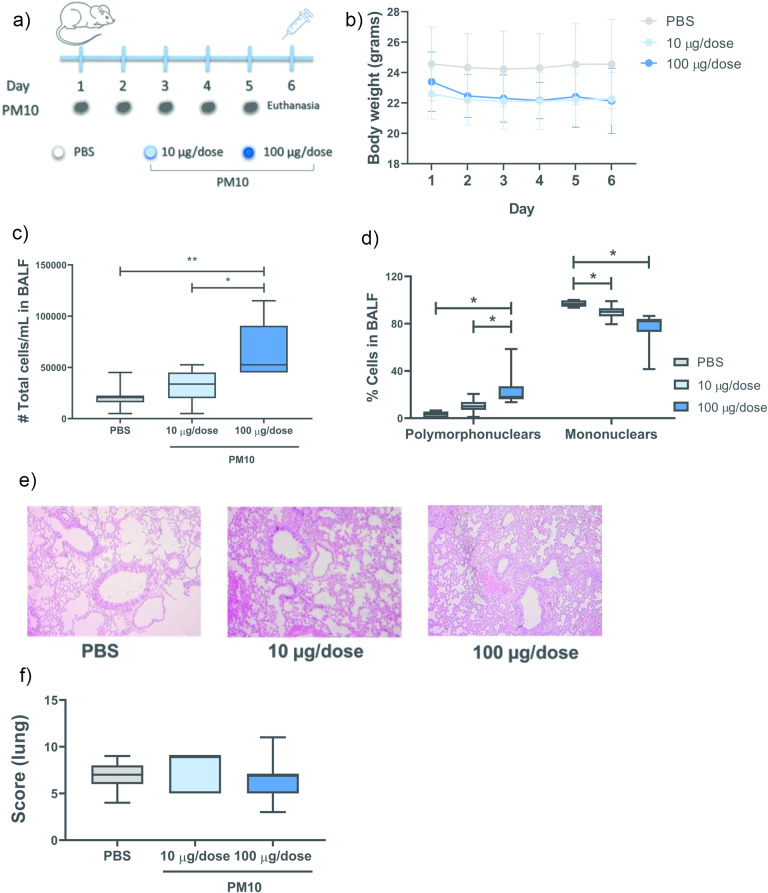
PM10 induces cell recruitment in the airways. Murine model of C57BL6 mice exposed to PM10 (10 and 100 µg/dose). **(A)** Scheme of exposure to PM10. **(B)** Record of body weight (in grams), **(C)** total cell count in BALF (total cells/mL), **(D)** differential count (% cells), **(E)** representative photographs of histological sections and **(F)** lung histopathology score. The data are presented as the median ± IQR (*n* = 6). Statistical comparison was made using the Kruskal-Wallis test with a confidence level of 95%, and Dunn’s HDS post hoc (or multiple reference points) tests were applied. Significant differences * *p* < 0.05; ** *p* < 0.01

The acute in vivo effects of PM10 were evaluated in male C57BL/6 mice intranasally exposed to PM10 (10 and 100 µg/dose) for 5 days. Euthanasia was performed on day 6 (Fig. 4A). Body weight changes were not observed during the experiment (Fig. 4B). A significant increase in cellularity was observed in the BALF from mice exposed to 100 µg/dose PM10 (Fig. 4C), mainly due to polymorphonuclear cells (Fig. 4D). However, no differences were observed in the lung histopathology analysis (Table S2, Fig. 4E and F).

### PM10 exposure induces an inflammatory response in the lungs

**Fig. 5:**
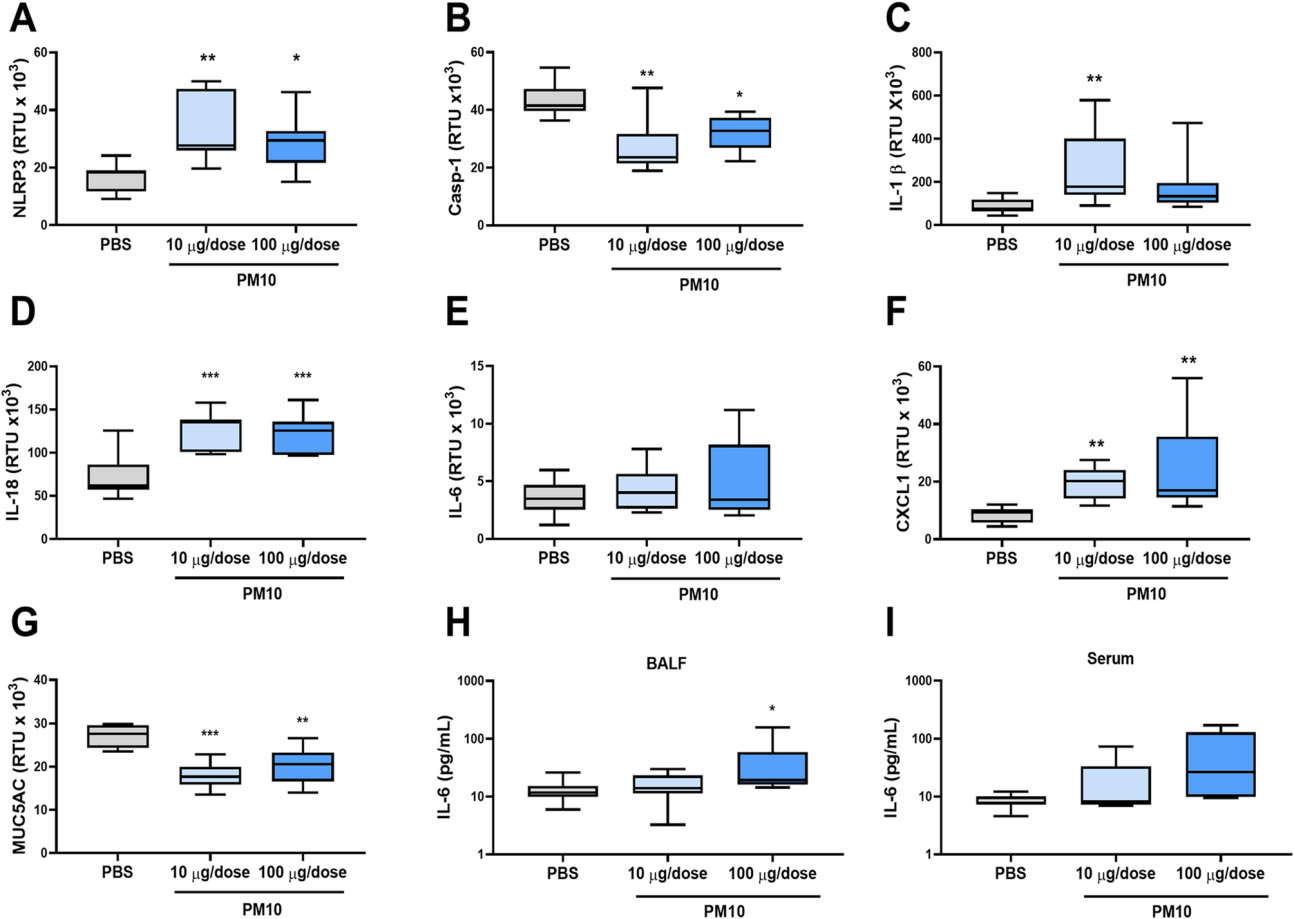
Exposure to PM10 induces an inflammatory response in the lungs. The gene expression of inflammatory components was quantified by qPCR from lung tissue of C57BL6 mice exposed to PM10. Results are presented as RTU (relative transcript units) x 10^3^ for **(A)** NLRP3, **(B)** Casp-1, **(C)** IL-1β, **(D)** IL-18, **(E)** IL-6, **(F)** CXCL1 and **(G)** MUC5AC and as pg/mL **(H)** IL-6 in BALF and **(I)** IL-6 in serum. Mice exposed to PBS were used as a negative control. The data were represented as median ± IQR (*n* = 6). Statistical comparison was made using the Kruskal-Wallis test with a confidence level of 95%, and Dunn’s HDS post hoc (or multiple reference points) tests were applied. Significant differences * *p* < 0.05; ** *p* < 0.01; ***p < 0.001

The mRNA of inflammatory genes was explored in the lungs. Significant increases in NLRP3 (*p* < 0.01), IL-1β (*p* < 0.01), IL-18 (*p* < 0.001) and CXCL1 (*p* < 0.01) was evidenced. Also, a reduction of Caspase-1 (*p* < 0.01) and MUC5AC (*p* < 0.001) was observed (Fig. 5A-F). Furthermore, gene expression was assessed in parabronchial nodules, but only an increase in IL-18 (*p* < 0.05) was observed (Figure S3). Finally, a significant increase in IL-6 was observed in BALF samples in response to 100 µg/dose PM10 but not in serum samples (Fig. 5H-I).

## Discussion

Particulate matter in the air is the most abundant pollutant, and is attributed to the most significant adverse effects on human health [25]. In addition, depending on its shape, aerodynamic diameter, and chemical composition, it can trigger different reactions when entering the respiratory tract [26]. Although the underlying mechanisms are still unclear, it has been proposed that oxidative stress, tissue damage, genotoxicity, vascular disruption, and inflammation mediate these harmful effects [16–18]. In particular, the cytotoxic and inflammatory response induced by PM10 exposure has been associated with exacerbating respiratory diseases such as asthma, COPD, lung cancer, and respiratory viral infections [2–4, 6].

In accordance, an increase in cytotoxicity was found in PBMCs exposed to PM10 present at the “Valle de Aburrá” in a dose-dependent manner. These results are similar to those reported by Van Den Heuvel et al., who reported a significant decrease in the viability of the bronchial epithelial cell line (BEAS2B) exposed to increasing concentrations of PM10, which was associated with the presence of heavy metals such as Cd and Pb. In addition, the same study reported a significant increase in the production of IL-8 associated with metals such as Cu, Ni and Zn [27]. In line with these results, in a systematic review that included studies between 1985 and 2020 carried out on lung epithelial cells, the authors found that the presence of transition metals and polycyclic aromatic hydrocarbons (PAH) correlates with the decrease in cell viability; even more, they reported a positive correlation between cellular cytotoxicity and the production of inflammatory mediators [28]. In agreement with these results, we previously reported the presence of different heavy metals within the PM10 sample [23], suggesting that these components possibly mediate the observed cytotoxic effects. Additionally, it is essential to highlight that most of the studies are carried out on lung epithelial cells, and this study reports that PM10 also has deleterious effects on cells of the immune system, which are essential players in the development of the immune response in lung tissue.

On the other hand, during PM exposure, there is a positive feedback between cytotoxic effects and inflammation and between the induction of reactive oxygen species, leading to a sustained response with adverse consequences [29]. In addition, it has been reported that exposure to PM induces an inflammatory process that involves the recruitment of cells to the lung [30]. In this regard, in our mouse model, we observed cell recruitment to the lung in response to PM10 exposure, with a significant increase in polymorphonuclear cells compared to those in unexposed mice. These results indicate that cells such as neutrophils are contributing to the inflammatory process in the lungs during acute exposure to PM10. However, it is important to consider that the recruitment of other cell populations such as tolerogenic dendritic cells, macrophages, and myeloid-derived suppressor cells (MDSC) that contribute to a poor environment has been reported in models of exposure to air pollutants for more than 8 days immunosuppressive in the lung [31]. Considering these findings, it is important to explore whether long-term exposure to PM10 present in the Aburrá Valley can induce the recruitment of other cell populations and how this could affect the development of respiratory diseases.

Moreover, although no significant differences in the histopathology analysis of the lungs were observed, a significant decrease in MUC5AC expression was found. Mucins are highly glycosylated macromolecules that make up the mucus present on the surface of airways and are a mechanism of immunity against pathogens and toxic substances; specifically, MUC5AC is secreted in the upper airways [32]. Contradictory results from exposure to air pollutants on the expression of this component have been previously reported. Similar to our results, Kania et al. reported a significant decrease in the expression of MUC5AC in Wistar rats subchronically exposed to coal dust [33]. In contrast, it has been reported that exposure to PM and diesel particles increases the production of this mucin in different cell lines [34–36]. These discrepancies could be due not only to the use of different models but also to the exposure time; considering that in murine models, the exposure was longer, it could be suggested that prolonged exposure can lead to a decrease in mucus components, altering the integrity of the epithelial barrier and mucociliary function, thus contributing to susceptibility to pathogens. Conversely, an increase in pro-inflammatory molecules expression was found in the lungs, indicating an inflammatory process in this tissue but not in the parabronchial nodules, suggesting a local inflammation.

Regarding the in vitro assays, PM10 was found to have the ability to induce the expression and production of pro-inflammatory cytokines such as IL-1β, IL-36γ, IL-6, IL-8, and TNF-α in PBMCs. Among these cytokines, IL-1β is a pleiotropic factor with multiple inflammatory effects that requires proteolytic maturation to acquire its bioactive form [37]. Cytosolic multiprotein platforms carry out this process, through so called inflammasomes that respond to microbial infections, endogenous danger signals, and environmental stimuli [38]. Although they are critical components for establishing an adequate inflammatory response, when there is an aberrant activation, they can contribute to the pathogenesis of various inflammatory diseases such as diabetes, cancer, and Alzheimer’s disease [39, 40]. In addition, it has been described that the activation of NLRP3 leads to pyroptosis and can also contribute to cell death through other pathways, such as apoptosis, necroptosis and ferroptosis [41], causing tissue damage.

In this regard, previous studies have shown that the NLRP3 inflammasome participates in the response to PM [20]. Here, it was found that, in addition to the significant induction of IL-1β mRNA, there was a downregulation of NLRP3, ASC, and IL-18 in in vitro assays. Regarding the negative regulation of some components, it has been previously described that basal expression of NRLP3 is sufficient for inflammasome activation, without the need for the transcription of additional components [42]. This could explain the observed decrease in NLRP3 and ASC mRNA levels, where PM10 could be inducing a rapid activation of the inflammasome, without inducing an increase in its gene expression. However, considering the increase in the levels of IL-1β mRNA and other cytokines, it would be important to consider whether there is a high rate of protein translation of NLRP3 and ASC and therefore an increase in the levels of these proteins, reflected in the decrease in mRNA. In this sense, it is necessary to carry out tests to corroborate this hypothesis. Likewise, the basal expression of IL-18 has been described in different cells such as PBMCs; even more, it has been reported that in the presence of some stimuli, protein production is detected without observing an increase in mRNA or even a significant decrease [42–45], which could also explain the observed decrease.

Additionally, the assembly of ASC complexes and the production of IL-1β were observed in THP-1 and PBMC supernatants, respectively, exposed to PM in a concentration-dependent manner. Furthermore, in a mouse model of exposure to PM, significant increases in the gene expression of NLRP3, IL-1β and IL-18 were found in the lung tissue. These results indicate that the NLRP3 inflammasome participates in the response triggered by PM10 present in the air of the “Valle de Aburrá”. According to these results, Jia et al. reported that inhibition of the activation of the NLRP3 pathway decreased lung damage, the inflammatory response, and the number of cells in BALF induced by in vivo exposure to PM2.5 [20]. Similarly, cigarette smoke-induced inflammation and pyroptosis death in epithelial cells through the activation of the NLRP3 inflammasome, processes that require ROS production [21]. Although there is still no consensus on the mechanisms that induce inflammasome assembly, potassium (K+) efflux is a joint event that triggers inflammasome activation in response to PM [46]. Consistent with this, glibenclamide (K + channel inhibitor) treatment significantly reduced IL-1β production in PM10-exposed PBMCs.

In a general context, it has been described that the development of lung diseases with a significant inflammatory component is characterized by the activation of resident epithelial cells and macrophages, leading to the recruitment and activation of neutrophils, monocytes, eosinophils, and lymphocytes. These recruited cells can produce ROS and inflammatory mediators that amplify the immune response, contributing to lung damage. This highlights the importance of evaluating the response induced by PM10 in epithelial cells and immune cells recruited to the lung, such as PBMCs. Furthermore, most studies evaluate the role of PM2.5 in activating the NLRP3 inflammasome since the most significant adverse effects have been attributed to this process. Among these studies, it has been described that exposure to PM2.5 induces the activation of the NLRP3 inflammasome in THP-1 cells, as well as the production of IL-1β and TGF-β1 in the BALF of mice and the significant deposition of collagen in the lower airways, suggesting inflammatory potential in pulmonary fibrosis [19]. PM2.5 has also been linked to the exacerbation of nasal mucosal damage in allergic rhinitis mice through NLRP3-mediated pyroptosis. They found that exposure to PM2.5 can aggravate rhinitis symptoms, promoting the secretion of serum IgE, and destroy the ultrastructure of the nasal mucosa. This change is accompanied by increases in NLRP3, casp-1, Gasdermina D (GSDMD) and IL-1β [47]. Although studies have focused on the relationship between the inflammasome and PM2.5 exposure, it is crucial to consider whether PM10 contributes to different lung diseases through this mechanism. Our evidence indicates that PM10 can activate the NLRP3 inflammasome in PBMCs, inducing IL-1β production and contributing to the triggered inflammatory response.

## Conclusion

The PM10 present in the Aburrá Valley decreases cell viability and induces the production of inflammatory mediators. Specifically, our results indicate that PM10 exposure in PBMCs induces an inflammatory response mediated by the NLRP3 inflammasome. Furthermore, acute exposure to PM10 induces the recruitment of cells to the lung, with a significant increase in polymorphonuclear cells. Similarly, it induces the expression of inflammatory mediators in lung tissue. These results indicate that although PM10 is deposited in the upper airways, the ability to trigger an inflammatory response and contribute to epithelial damage can exacerbate diseases such as fibrosis and rhinitis and increase susceptibility to infections. Therefore, it is necessary to design environmental strategies to reduce emission levels and protect people’s health.

### Electronic supplementary material

Below is the link to the electronic supplementary material.

Supplementary Material 1

Supplementary Material 2

## Data Availability

All data generated or analyzed during this study are included in this published article [and its supplementary information files].

## Declarations

### Disclaimers

The opinions expressed in this article are our own and do not constitute the institution’s official position.

## References

[1] Fuller R (2022). Pollution and health: a progress update. Lancet Planet Health.

[2] Doiron D, et al. Air pollution, lung function and COPD: results from the population-based UK Biobank study. Eur Respir J. 2019;54. 10.1183/13993003.02140-2018. 10.1183/13993003.02140-2018 31285306

[3] Hamra GB (2014). Outdoor particulate matter exposure and lung cancer: a systematic review and meta-analysis. Environ Health Perspect.

[4] Hei Collaborative Working Group on Air Pollution P (2012). Effects of short-term exposure to air pollution on hospital admissions of young children for acute lower respiratory infections in Ho Chi Minh City, Vietnam. Res Rep Health Eff Inst.

[5] Mishra R, Krishnamoorthy P, Gangamma S, Raut AA, Kumar H (2020). Particulate matter (PM10) enhances RNA virus infection through modulation of innate immune responses. Environ Pollut.

[6] Tecer LH, Alagha O, Karaca F, Tuncel G, Eldes N (2008). Particulate matter (PM(2.5), PM(10 – 2.5), and PM(10)) and children’s hospital admissions for asthma and respiratory diseases: a bidirectional case-crossover study. J Toxicol Environ Health A.

[7] Marín-Palma D, et al. PM10 promotes an inflammatory cytokine response that may impact SARS-CoV-2 replication in vitro. Front Immunol. 2023;14. 10.3389/fimmu.2023.1161135. 10.3389/fimmu.2023.1161135 PMC10166799 37180105

[8] Katanoda K (2011). An association between long-term exposure to ambient air pollution and mortality from lung cancer and respiratory diseases in Japan. J Epidemiol.

[9] Raaschou-Nielsen O (2013). Air pollution and lung cancer incidence in 17 European cohorts: prospective analyses from the European study of cohorts for Air Pollution effects (ESCAPE). Lancet Oncol.

[10] Gómez-Gallego DM, Hernández JC (2022). Mendivil-De La Ossa, J. A. Efectos adversos de la exposición prenatal Al material particulado Del aire Sobre El Feto Y El recién nacido. Iatreia.

[11] Heusinkveld HJ (2016). Neurodegenerative and neurological disorders by small inhaled particles. Neurotoxicology.

[12] Nasser Z (2015). Outdoor particulate matter (PM) and associated cardiovascular diseases in the Middle East. Int J Occup Med Environ Health.

[13] Castanheiro A (2021). Morphological and elemental characterization of leaf-deposited particulate matter from different source types: a microscopic investigation. Environ Sci Pollut Res Int.

[14] Steenhof M (2011). In vitro toxicity of particulate matter (PM) collected at different sites in the Netherlands is associated with PM composition, size fraction and oxidative potential–the RAPTES project. Part Fibre Toxicol.

[15] Thomson EM (2015). Cytotoxic and inflammatory potential of size-fractionated particulate matter collected repeatedly within a small urban area. Part Fibre Toxicol.

[16] Cevallos VM, Diaz V, Sirois CM (2017). Particulate matter air pollution from the city of Quito, Ecuador, activates inflammatory signaling pathways in vitro. Innate Immun.

[17] Piao MJ (2018). Particulate matter 2.5 damages skin cells by inducing oxidative stress, subcellular organelle dysfunction, and apoptosis. Arch Toxicol.

[18] Valderrama A (2022). Particulate matter (PM10) induces in vitro activation of human neutrophils, and lung histopathological alterations in a mouse model. Sci Rep.

[19] Zheng R (2018). NLRP3 inflammasome activation and lung fibrosis caused by airborne fine particulate matter. Ecotoxicol Environ Saf.

[20] Jia H (2021). PM2.5-induced pulmonary inflammation via activating of the NLRP3/caspase-1 signaling pathway. Environ Toxicol.

[21] Zhang MY (2021). Cigarette smoke extract induces pyroptosis in human bronchial epithelial cells through the ROS/NLRP3/caspase-1 pathway. Life Sci.

[22] Liu W (2022). Particulate matter pollution and asthma mortality in China: a nationwide time-stratified case-crossover study from 2015 to 2020. Chemosphere.

[23] Marin-Palma D (2023). Physicochemical characterization and evaluation of the cytotoxic effect of Particulate Matter (PM10). Water Air Soil Pollut.

[24] Marin-Palma D, et al. Curcumin inhibits in Vitro SARS-CoV-2 infection in Vero E6 cells through multiple antiviral mechanisms. Molecules. 2021;26. 10.3390/molecules26226900. 10.3390/molecules26226900 PMC8618354 34833991

[25] Stanaway JD (2018). Global, regional, and national comparative risk assessment of 84 behavioural, environmental and occupational, and metabolic risks or clusters of risks for 195 countries and territories, 1990–2017: a systematic analysis for the global burden of Disease Study 2017. Lancet.

[26] Shao J (2018). The pro-inflammatory effects of particulate matter on epithelial cells are associated with elemental composition. Chemosphere.

[27] Van Den Heuvel R (2016). Identification of PM10 characteristics involved in cellular responses in human bronchial epithelial cells (Beas-2B). Environ Res.

[28] Kermani M (2021). Potential cytotoxicity of trace elements and polycyclic aromatic hydrocarbons bounded to particulate matter: a review on in vitro studies on human lung epithelial cells. Environ Sci Pollut Res.

[29] Valacchi G, Magnani N, Woodby B, Ferreira SM, Evelson P (2020). Particulate matter induces tissue OxInflammation: from mechanism to damage. Antioxid Redox Signal.

[30] Happo MS (2010). Inflammation and tissue damage in mouse lung by single and repeated dosing of urban air coarse and fine particles collected from six European cities. Inhal Toxicol.

[31] Colarusso C (2019). The inhibition of Caspase-1- does not revert particulate matter (PM)-Induced Lung Immunesuppression in mice. Front Immunol.

[32] Rose MC, Voynow JA (2006). Respiratory tract mucin genes and mucin glycoproteins in health and disease. Physiol Rev.

[33] Kania N (2014). Subchronic inhalation of coal dust particulate matter 10 induces bronchoalveolar hyperplasia and decreases MUC5AC expression in male Wistar rats. Exp Toxicol Pathol.

[34] Kim SS (2017). Airborne particulate matter increases MUC5AC expression by downregulating Claudin-1 expression in human airway cells. BMB Rep.

[35] Na HG, Kim YD, Choi YS, Bae CH, Song SY (2019). Diesel exhaust particles elevate MUC5AC and MUC5B expression via the TLR4-mediated activation of ERK1/2, p38 MAPK, and NF-kappaB signaling pathways in human airway epithelial cells. Biochem Biophys Res Commun.

[36] Shin SH, Ye MK, Lee DW, Kang BJ, Chae MH. Effect of Korean Red Ginseng and Rg3 on Asian Sand Dust-Induced MUC5AC, MUC5B, and MUC8 expression in bronchial epithelial cells. Molecules. 2021;26. 10.3390/molecules26072002. 10.3390/molecules26072002 PMC8037637 33916022

[37] Lopez-Castejon G, Brough D (2011). Understanding the mechanism of IL-1beta secretion. Cytokine Growth Factor Rev.

[38] Jin C, Flavell RA (2010). Molecular mechanism of NLRP3 inflammasome activation. J Clin Immunol.

[39] Wang L, Hauenstein AV (2020). The NLRP3 inflammasome: mechanism of action, role in disease and therapies. Mol Aspects Med.

[40] Tabares-Guevara JH, Villa-Pulgarin JA, Hernandez JC (2021). Atherosclerosis: immunopathogenesis and strategies for immunotherapy. Immunotherapy.

[41] Zheng M, Kanneganti TD (2020). The regulation of the ZBP1-NLRP3 inflammasome and its implications in pyroptosis, apoptosis, and necroptosis (PANoptosis). Immunol Rev.

[42] Lin KM (2014). IRAK-1 bypasses priming and directly links TLRs to rapid NLRP3 inflammasome activation. Proc Natl Acad Sci U S A.

[43] Manfrere KCG, et al. Imbalanced IL-1B and IL-18 expression in Sezary Syndrome. Int J Mol Sci. 2023;24. 10.3390/ijms24054674. 10.3390/ijms24054674 PMC10003479 36902104

[44] Moller B (2001). Expression of interleukin-18 and its monokine-directed function in rheumatoid arthritis. Rheumatology (Oxford).

[45] Zhu Q, Kanneganti TD (2017). Cutting Edge: distinct Regulatory mechanisms Control Proinflammatory cytokines IL-18 and IL-1beta. J Immunol.

[46] Munoz-Planillo R (2013). K(+) efflux is the common trigger of NLRP3 inflammasome activation by bacterial toxins and particulate matter. Immunity.

[47] Li J (2021). Fine particulate matter exposure exacerbated nasal mucosal damage in allergic rhinitis mice via NLRP3 mediated pyroptosis. Ecotoxicol Environ Saf.

