# Supplementary figures and images for: Coarse particulate matter (PM10) induce an inflammatory response through the NLRP3 activation
Source: J Inflamm (Lond). 2024 May 2;21:15. doi: 10.1186/s12950-024-00388-9 (PMC11064351; doi:10.1186/s12950-024-00388-9)

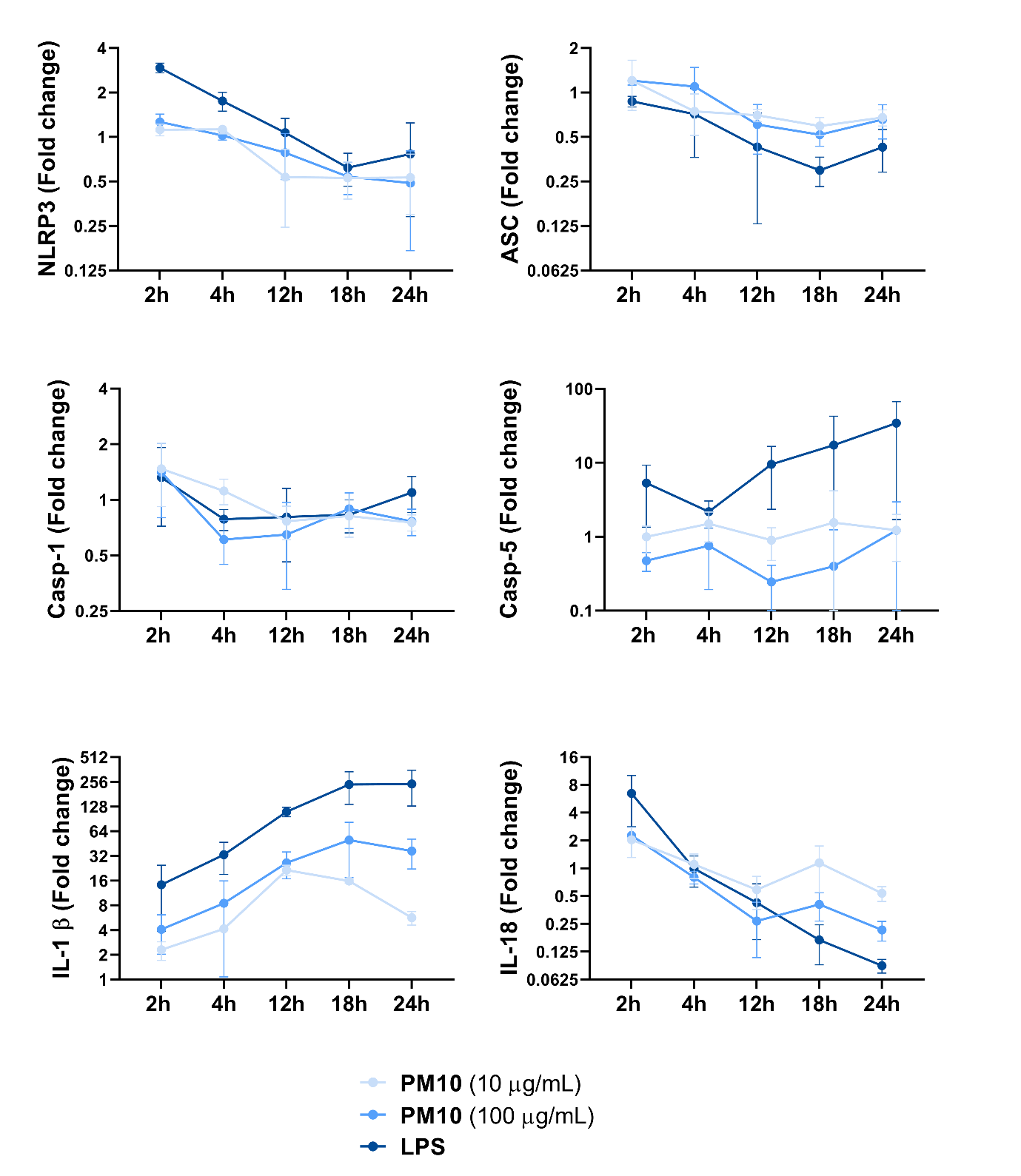

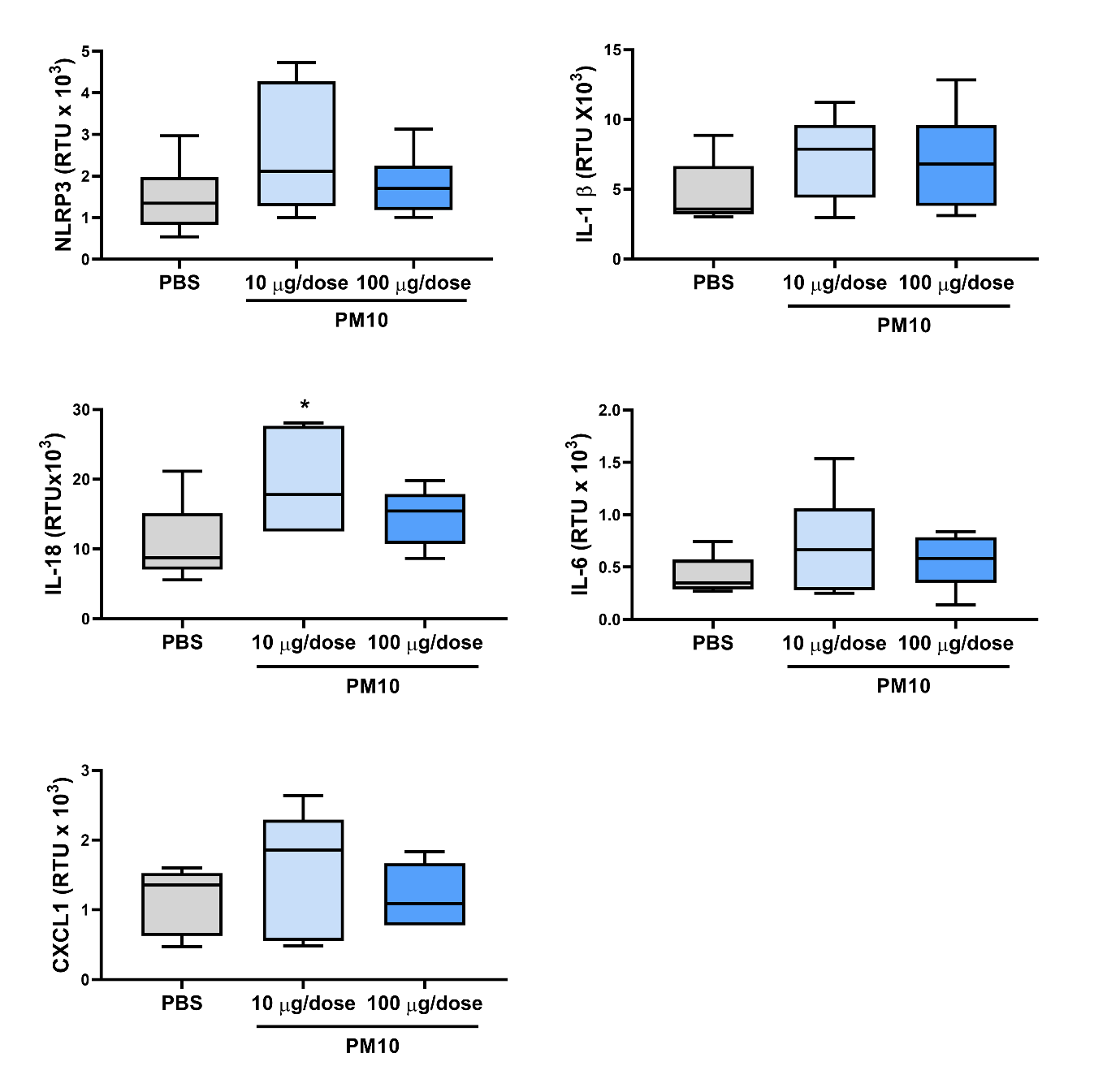

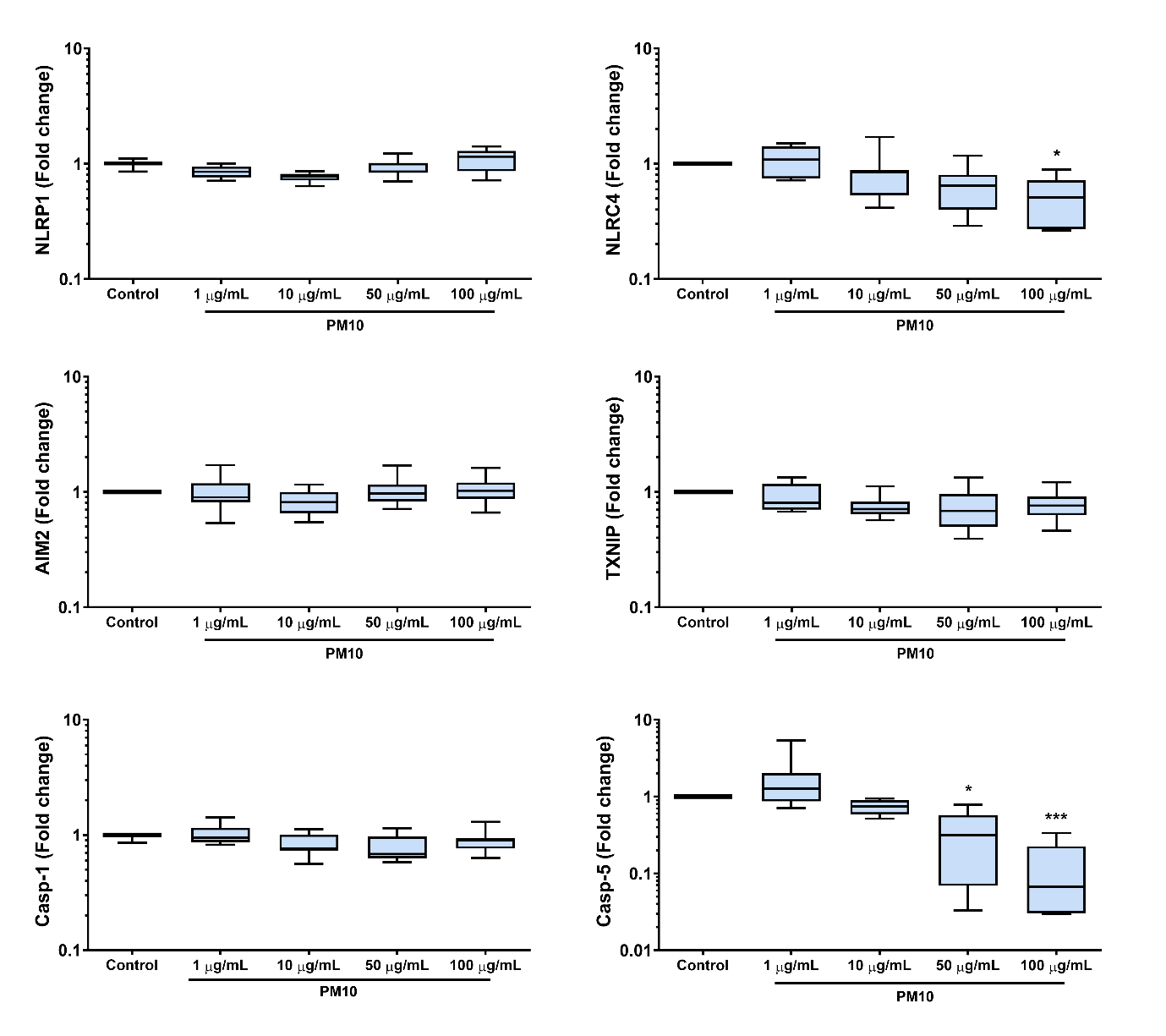

Supplement: Supplementary file 1 — Supplementary Material 1 [file 12950_2024_388_MOESM1_ESM.docx]
