# Supplementary material for: Coarse particulate matter (PM10) induce an inflammatory response through the NLRP3 activation
Source: J Inflamm (Lond). 2024 May 2;21:15. doi: 10.1186/s12950-024-00388-9 (PMC11064351; doi:10.1186/s12950-024-00388-9)
Supplement: Supplementary file 2 — Supplementary Material 2 [file 12950_2024_388_MOESM2_ESM.docx]

**Supplementary Figures and Tables**

**Supplementary Figures**

**Figure S1. Kinetics of gene expression of inflammatory components in PBMCs exposed to PM10.** Gene expression of inflammatory components was quantified by qPCR from PBMCs exposed to PM10 (10 and 100 µg/mL) at different times (2-24h). LPS (0.05 µg/mL) was used as a positive control. Data were presented as mean ± SD (n=3) and in a pooled plot.


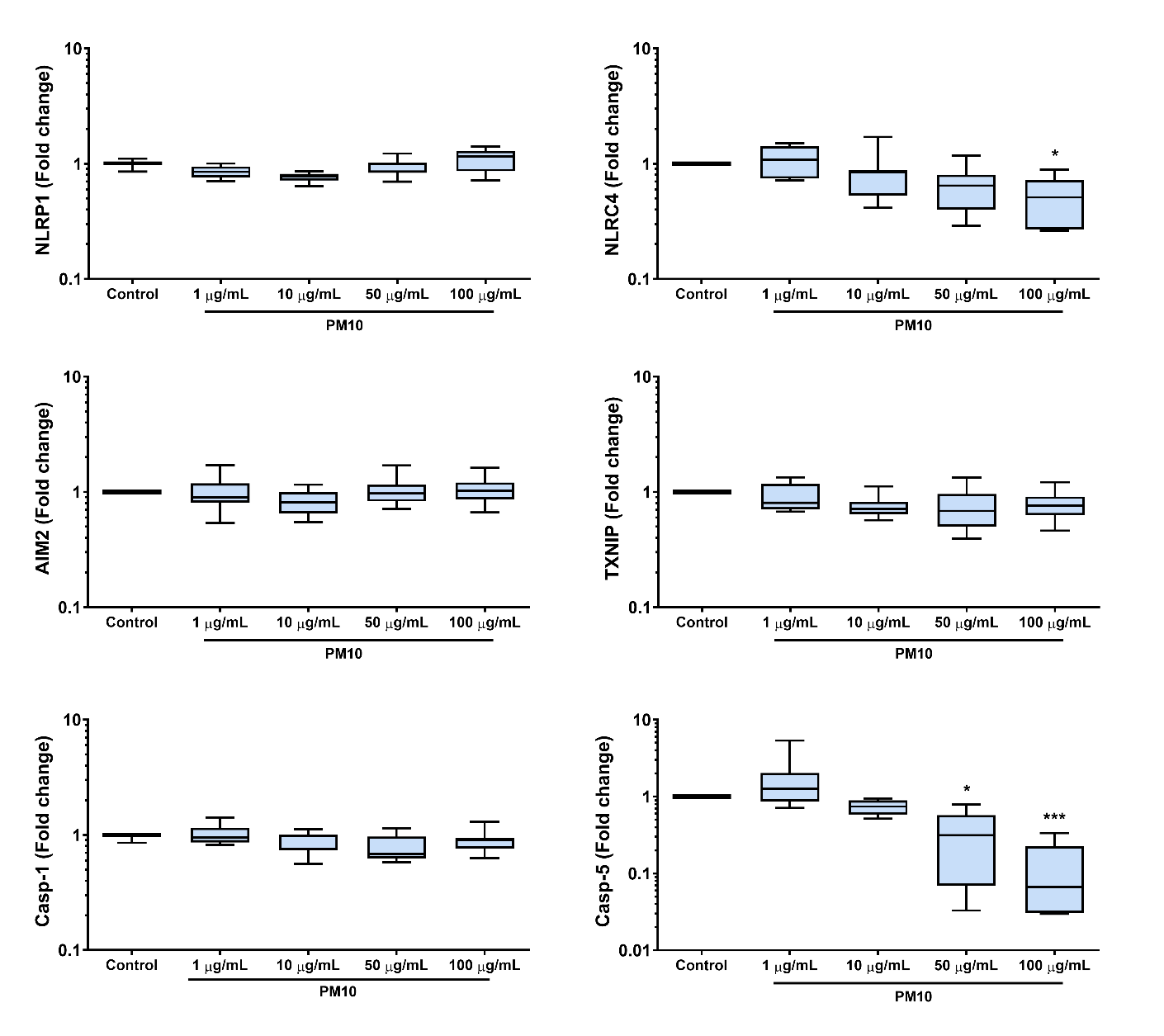


**Figure S2. PM10 alters the expression of inflammasome components.** Gene expression of inflammasome components was quantified by qPCR in PBMCs exposed for 18h to increasing concentrations of PM10 (1-100 µg/mL). Results are presented as fold changes of NLRP1, NLRC4, AIM2, TXNIP, Caspase-1 and Caspase-5. Data are presented as median ± IQR (n=7). Statistical comparison was made using Kruskal-Wallis tests with a confidence level of 95%, and Dunn's HDS post hoc (or multiple benchmarks) tests were performed. Significant differences are represented at the top of the bars (*p < 0.05; ***p < 0.001).

**Figure S3. Gene expression of inflammatory components in parabronchial nodules.** Gene expression of inflammatory components was quantified by qPCR from parabronchial tissue of C57BL6 mice exposed to PM10. Results are presented as RTU x 10^3^ of NLRP3, IL-1β, IL-18, IL-6 and CXCL1. Naive mice were used as a negative control. Data were represented as median ± IQR (n = 6). Statistical comparison was made using the Kruskal-Wallis test with a confidence level of 95%, and Dunn's HDS post hoc (or multiple reference points) tests were applied. Significant differences * p<0.05.

**Supplementary Tables**

**Table S1. Primers for human genes**

| **Gene** | **Primers 5´-3´** | **Annealing temperature (°C)** |
| --- | --- | --- |
| **Human genes** | | |
| **IL-1β** | Fw: GGATATGGAGCAACAAGTGG  Rv: ATGTACCAGTTGGGGAACTG | 60 |
| **IL36γ** | Fw: TAGGACCTCCACCCTTGAGTC  Rv: AATGATGGGCTGGTCTCTCTT | 60 |
| **IL-6** | Fw: GGGGTGGTTATTGCATC  Rv: ATTCGGTACATCCTCGAC | 56 |
| **IL-8** | Fw: ACTGAGAGTGATTGAGAGTGGAC  Rv: AACCCTCTGCACCCAGTTTTC | 60 |
| **IL-18** | Fw: ATGGCTGCTGAACCAGTAGAAG  Rv: CAGCCATACCTCTAGGCTGGC | 62 |
| **TNF-α** | Fw: GGCTCCAGGCGGTGCTTGTTC  Rv: AGACGGCGATGCGGCTGATG | 60 |
| **NLRP1** | Fw: CTATACTTCCCGAGGCATCCTT  Rv: GGTCTTGGAAGTCAGTGTGAGT | 56 |
| **NLRP3** | Fw: AGCACCAGCCAGAGTCTAAC  Rv: CCCCAACCACAATCTCCGAAT | 57 |
| **NRLC4** | Fw: CTCTCATGGTGGAAGCCAGTCC  Rv: ACAGAGACTTGACTATGTAATCC | 56 |
| **AIM2** | Fw: AAGCGCTGTTTGCCAGTTAT  Rv: CACACGTGAGGCGCTATTTA | 55 |
| **TXNIP** | Fw: CCAGCCAACTCAAGAGACA  Rv: GCCCATCAGGAATGAACA | 60 |
| **ASC** | Fw: AACCCAAGCAAGATGCGGAAG  Rv: TTAGGGCCTGGAGGAGCAAG | 62 |
| **Caspase 1** | Fw: CAAGGGTGCTGAACAAGG  Rv: GGGCATAGCTGGGTTGTC | 60 |
| **Caspase 5** | Fw: TCATTTGAAGTTCCACAGGCTA  Rv: TGCCTGTGGTTTCATTTTCA | 60 |
| **PGK** | Fw: GTTGACCGAATCACCGACC  Rv: CGACTCTCATAACGACCCGC | 60 |
| **Mouse genes** | | |
| **NLRP3** | Fw: GACCAGCCAGAGTGGAATGAC  Rv: CTGCGTGTAGCGACTGTTGAG | 60 |
| **Casp-1** | Fw: CTTGGAGACATCCTGTCAGGG  Rv: AGTCACAAGACCAGGCATATTCT | 60 |
| **IL-1β** | Fw: TCGCTCAGGGTCACAAGAAA  Rv: CATCAGAGGCAAGGAGGAAAAC | 58.3 |
| **IL-18** | Fw: TGGTGGGGGTTCTCTGTGGTT  Rv: TTGAGGCGGCTTTCTTTGTCC | 61.9 |
| **CXCL1 (KC)** | Fw: TGGCTGGGATTCACCTCAAG  Rv: CCGTTACTTGGGGACACCTT | 61.4 |
| **IL-6** | Fw: TCTATACCACTTCACAAGTCGGA  Rv: GAATTGCCATTGCACAACTCTTT | 60 |
| **MUC5AC** | Fw: CTGTGACATTATCCCATAAGCCC  Rv: AAGGGGTATAGCTGGCCTGA | 60 |
| **GADPH** | Fw: AGGTCGGTGTGAACGGATTTG  Rv: TGTAGACCATGTAGTTGAGGTCA | 60 |

| **Score** | **Atelectasia** | **Hemorrhage** | **Edema** | **Emphysema** | **Congestion** | **Pigments** | **PNN** | **Lymphocytes** |
| --- | --- | --- | --- | --- | --- | --- | --- | --- |
| 0 | Absent | No presence of erithrocytes in alveoles | Absent | Absent | Absent | Absent | No presence of PNN in alveoles or bronchioles | No presence of lymphocytes in alveoles or bronchioles |
| 1 | Limited | Limited presence of erithrocytes in alveoli | Minimal swelling of alveolar walls | Limited | Limited | Limited | limited increase of PNN in alveoli and /or bronchioles | limited increase of lymphocytes in alveoli and /or bronchioles |
| 2 | Moderated | moderated presence of erithrocytes in alveolis | Moderated swelling of alveolar walls | Moderated | Moderated | Moderated | Moderated increase of PNN in alveoli and /or bronchioles | Moderated increase of lymphocytes in alveoli and /or bronchioles |
| 3 | Elevated | Elevated presence of erithrocytes in alveolis | Severe swelling of alveolar walls | Severe | Severe | Elevated | Elevated increase of PNN in alveoli and /or bronchioles | Elevated increase of lymphocytes in alveoli and /or bronchioles |

**Table 2: Histopathological score of tissue**.0=Absent or normal. 1= Limited 2= Moderated. 3= Severe
